# RNA Polymerase 1 inhibitors against African trypanosomes *in vitro* and in mice

**DOI:** 10.1128/aac.01492-25

**Published:** 2026-03-27

**Authors:** Alexander V. Cumming, Nathaniel P. Nenortas, Elizabeth Nenortas, Rahul P. Bakshi, Emily R. Caton, Estefany Rios-Guzman, Mary E. Barry, James C. Barrow, Marikki Laiho, Theresa A. Shapiro

**Affiliations:** 1 Division of Clinical Pharmacology, Department of Medicine, The Johns Hopkins University School of Medicine 1500 , Baltimore, Maryland, USA; 2 Department of Pharmacology, Lieber Institute for Brain Development, The Johns Hopkins University School of Medicine 1500 , Baltimore, Maryland, USA; 3 Department of Radiation Oncology and Molecular Radiation Sciences, The Johns Hopkins University School of Medicine 1500 , Baltimore, Maryland, USA; 4 Department of Physiology, Pharmacology and Therapeutics, The Johns Hopkins University School of Medicine 1500 , Baltimore, Maryland, USA; The Children's Hospital of Philadelphia, Philadelphia, Pennsylvania, USA

**Keywords:** *Trypanosoma brucei brucei*, trypanosomiasis, RNA Polymerase 1, inhibitors, hollow fiber model system, PK-PD, BMH-21, LI-623, BOB-42

## Abstract

Therapy of human African trypanosomiasis, fatal if not treated, has been greatly improved by orally available and safer fexinidazole, but reliable cure of late-stage central nervous system (CNS) infection remains a challenge. African trypanosomes are unique among eukaryotes in having an RNA Polymerase 1 that transcribes not only rRNA but also mRNA that encodes the abundant variable surface glycoprotein (VSG) that coats the cell membrane of bloodstream-form parasites and allows them to evade host immune defenses. RNA Pol 1 inhibitor BMH-21, an intercalator not associated with DNA damage and a lead for new cancer therapy, has reported activity against the synthesis of rRNA and VSG mRNA in *Trypanosoma brucei brucei*. We evaluated a library of BMH-21 analogs against bloodstream-form *T. b. brucei in vitro* and found limited tolerance for structural modification. Striking and time-dependent bimodal dose-response curves indicate these compounds have a complex mechanism of action. Most potent against *T. b. brucei* was benzopyridoquinazoline Compound **2**, with an EC_50_ of 84 nM. Analysis of *in vitro* pharmacokinetic-pharmacodynamic (PK-PD) relationships by a hollow fiber model system revealed that in *T. b. brucei*, the kinetic driver of Compound **2** is drug concentration, a finding that was confirmed by dose fractionation in infected mice and that highlights the utility of the *in vitro* system for assessing antitrypanosomal PK-PD. Although Compound **2** accumulates 20-fold in mouse brain tissue vs plasma, efficacy against CNS *T. b. brucei* in mice was limited. RNA Pol 1 remains an attractive target for developing new and more selectively toxic antitrypanosomal agents.

## INTRODUCTION

African trypanosomes, transmitted in sub-Saharan Africa by the tsetse fly, cause human African trypanosomiasis (HAT, sleeping sickness) and nagana in animals (1–3). In humans, the disease transitions from an initial systemic infection to a meningoencephalitis that is fatal if not treated. Infection with *Trypanosoma brucei gambiense* may take years to progress, whereas the more virulent *T. b. rhodesiense* causes death in weeks to months. Closely related *T. b. brucei* is similarly lethal in animals and poses an economic burden in Africa, but it is not infectious to humans and thus provides a safe and useful lab reagent. FDA-approved in 2021, orally dosed fexinidazole has revolutionized the therapy of *T. b. gambiense* infections, which previously required repeated parenteral dosing with toxic drugs (4, 5). However, fexinidazole is not reliably active against central nervous system (CNS) parasites (6, 7), and CNS *T. b. rhodesiense* infections in particular may require repeated parenteral doses of melarsoprol, a toxic trivalent organic arsenical that itself kills ~6% of treated patients. Several experimental agents are in development (8, 9), but there remains a need for safe new oral therapies that work reliably against both peripheral and CNS infections.

Trypanosomes are phylogenetically ancient eukaryotes (10)—flagellated protozoans—that have distinctly unusual structural and metabolic features. Antigenic variation allows these extracellular pathogens to avoid host immune defenses and thus precludes vaccine development (11, 12). African trypanosomes are uniformly coated with 10^7^ copies of a single variable surface glycoprotein (VSG) that is regularly switched to prevent detection. Uniquely in trypanosomes, RNA Polymerase 1 (Pol 1) transcribes not just rRNA but also the mRNA that codes for the abundant VSG proteins (13). Inhibition of VSG synthesis leads to cell cycle arrest, and trypanosomes with a compromised VSG coat are cleared by the immune system (14). Also distinctive in trypanosomes is their mitochondrial DNA, a mass of interlocked DNA maxicircles (~25 kb) and minicircles (~1 kb), visible by light microscopy, known as the kinetoplast (15, 16). Encoded by kinetoplast DNA are genes for the mitochondrial rRNAs and for proteins that participate in bioenergetics. Mature RNAs are obtained by a complex and bizarre process entailing the use of minicircle-encoded guide RNAs to insert or delete U residues in an immature maxicircle-encoded transcript. RNAs in trypanosome mitochondria are reportedly synthesized by a phage-like mtRNAP (17, 18).

Focus on RNA Pol 1 as a promising target for anticancer drug development originated with the identification of BMH-21 (benzopyridoquinazoline Compound **1** in Table 1) in a screen for p53 pathway activation (19). The compound was subsequently found to intercalate into GC-rich regions in rDNA (including G-quadruplex structures; see reference 20), repress RNA Pol 1 transcription, and induce degradation of the RPA194 subunit of RNA Pol 1, effects obtained without DNA damage (19, 21, 22). Interference with RNA Pol 1 activity kills rapidly dividing malignant cells that are overly dependent on it to meet the demands for brisk protein synthesis (23). In an effort to improve antitumor potency and selectivity, a library of analogs was synthesized and tested, revealing that activity can be improved, albeit within a clearly limited chemical space (24, 25). Unusually and of considerable importance for antitrypanosomal therapy, the lead from that screen, LI-623 (or BOB-42 [26], Compound **2** in Table 1), accumulates nearly 20-fold in brain tissue relative to plasma (Fig. S1).

**TABLE 1:** Structure-activity of RNA Pol I inhibitors against *T. b. brucei* MiTat 1.2 *in vitro*

Compound	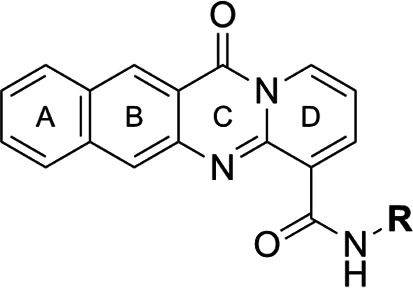 Scaffold A		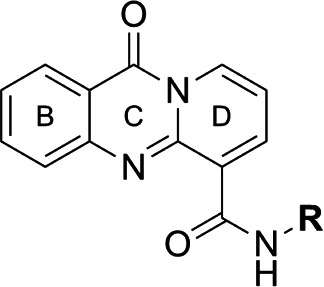 Scaffold B
	Scaffold	R	EC_50_ (μM)^*a*^
**1** (BMH-21)	A	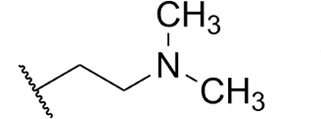	0.11
**2**	A	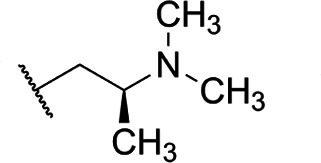	0.084
**3**	A	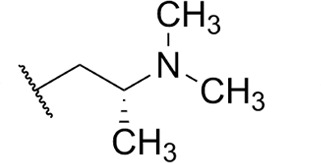	0.46
**4**	A	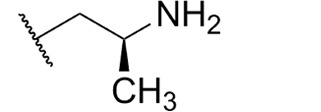	0.23
**5**	A	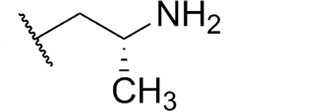	0.52
**6**	A	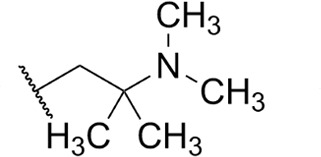	0.22
**7**	A	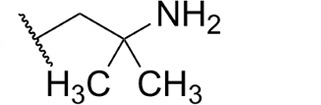	0.35
**8**	A	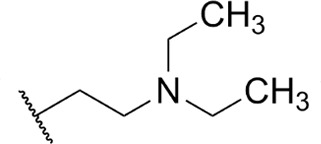	1.2^*b*^
**9**	A	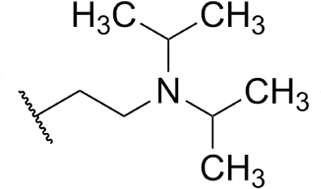	2.0
**10**	A	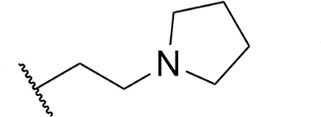	0.17
**11**	B	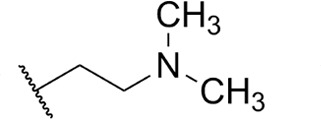	2.5^*b*^
**12**	B	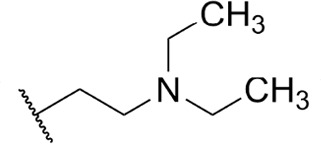	2.7^*b*^

^*a*^ Overall EC_50_ values for biphasic curve fit; all *R*^2^ values ≥0.994; CV for all data points ≤3.9. Unless indicated otherwise, data represent the mean of two or more biological replicates, each conducted in quadruplicate.

^*b*^ Data from one experiment in quadruplicate.

A previous study of RNA Pol 1 inhibitors (including BMH-21) revealed selective toxicity against *T. b. brucei*, with submicromolar EC_50_s, a fall in rRNA and VSG RNA levels, and disruption of the subnuclear compartments where rRNA and VSG are synthesized (27).

In this work, we further explored the possible repurposing of experimental antitumor RNA Pol 1 inhibitors for use against African trypanosomes. All experiments were conducted with *T. b. brucei* or *T. b. gambiense*. Structure-activity analyses characterized antitrypanosomal potency and selective toxicity, and lead Compound **2** was tested in combination with other agents. Compound **2** was then evaluated in an *in vitro* hollow fiber model system to identify the pharmacokinetic driver of its action against *T. b. brucei*, which was confirmed in trypanosome-infected mice. Efficacy in early- and late-stage murine infection was also evaluated.

## RESULTS

### RNA Polymerase 1 inhibitors against protozoans and mammalian cells *in vitro*

**Fig 1:**
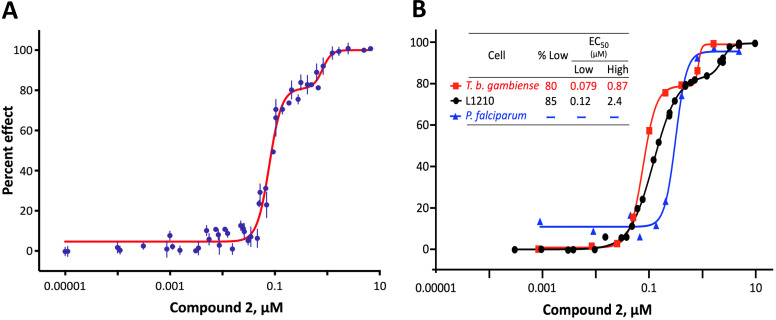
Cytotoxicity of Compound **2** against protozoans and mammalian cells. (**A**) Dose-response against *T. b. brucei* MiTat 1.2. When data are fit to a biphasic curve, two contributions to efficacy are evident: one with a low EC_50_ of 0.075 µM that provides 79% of the overall efficacy and a second with a 10-fold higher EC_50_ of 0.75 µM. Overall EC_50_ (concentration to obtain 50% effect on the entire biphasic curve) was 0.084 µM. Depicted are M ± SD (all CVs ≤3.9), mean *R*^2^ 0.997, from 10 biologically independent experiments, each concentration in quadruplicate. (**B**) Compound **2** against human pathogens *T. b. gambiense* and *Plasmodium falciparum,* or L1210 mammalian cells, as indicated. Overall EC_50_ values were 0.092, 0.31, and 0.15 μM, respectively. All CVs ≤8.8 and *R*^2^ fits ≥0.981.

We investigated the cytotoxicity of parent compound BMH-21 (Compound **1**, Table 1) and several dozen previously described structural analogs (24) against bloodstream-form *T. b. brucei* and found that the activity was distinctly biphasic (Fig. 1A). For the most potent analog (Compound **2**), 79% of efficacy was provided by a low EC_50_ target (0.075 µM) and the balance by a less susceptible one (0.75 µM). Biphasic responses with similar ratios (~80:20, low:high) were also obtained for Compound **2** against *T. b. gambiense* and L1210 mouse lymphocytic leukemia cells, but activity against *Plasmodium falciparum* malaria parasites appeared to be monophasic (Fig. 1B). Potency of Compound **2** was greatest against African trypanosomes (0.084–0.092 µM) and least against mammalian cells (0.31 µM); the therapeutic index for trypanosomes vs mammalian cells was ≤3. The latter may be an underestimate of selective toxicity, given the assayed mammalian line was L1210 lymphocytic leukemia cells and demand for RNA Pol 1 activity is high in rapidly dividing malignant cells, including leukemias (23, 26).

### Structure-activity against African trypanosomes

BMH-21 (**1**) and its analogs were tested for activity against *T. b. brucei* in our standard 24-h assay (Table 1; Table S1). Of the 16 compounds for which complete dose-response curves could be obtained, 15 were clearly biphasic and one was ambiguous. Notably, across a nearly 30-fold range of compound potencies, the contribution of each phase to overall efficacy remained essentially constant (low EC_50_ fraction, 82% ± 3%). The ratio of high to low EC_50_ values was also remarkably constant (7.9 ± 1.9). Given the small differences among compounds in these characteristics, and in order to simplify and focus the following structure-activity analysis, the EC_50_ values compiled in Table 1 and Table S1 were the overall single values obtained from a biphasic curve fit. On average, they vary less than 10% from the cognate low EC_50_.

Although clear structural patterns could be discerned among the analogs (Table 1), only one modification to BMH-21 (**1**), the *S*-enantiomer of methyl added adjacent to the terminal amine (Compound **2**), provided a marginal increase in activity (*P* = 0.014). Interestingly, Compound **2** was significantly more potent than its *R*-isomer (**3**, *P* <0.0001), a finding reproduced for similar configurations on a primary amine side chain (**4** vs **5**, *P* ≤ 0.05). Dimethyl substitution adjacent to or beta to the terminal amine reduced activity (**6** vs **2** or **7** vs **4**, respectively). Increasing the size of aliphatic residues on the terminal amine systematically reduced potency (**8** or **9** vs **1**), although constraining the terminus to a pyrrolidine increased activity sevenfold (**10** vs **8**). Importance of the four-member aromatic ring system was evidenced by loss of activity for analogs missing the benzo A ring (**11** vs **1** or **12** vs **8**). Other modifications of Compound **1** (Table S1), including bulky or aromatic residues in the side chain (**13**-**26**), further substitution of the quinazoline carboxamide (**27**-**33**), or conversion of carboxamide to carboxylate (**34**), all reduced activity.

### Possible contributions to biphasic dose-response curve

#### Time dependence

**Fig 2:**
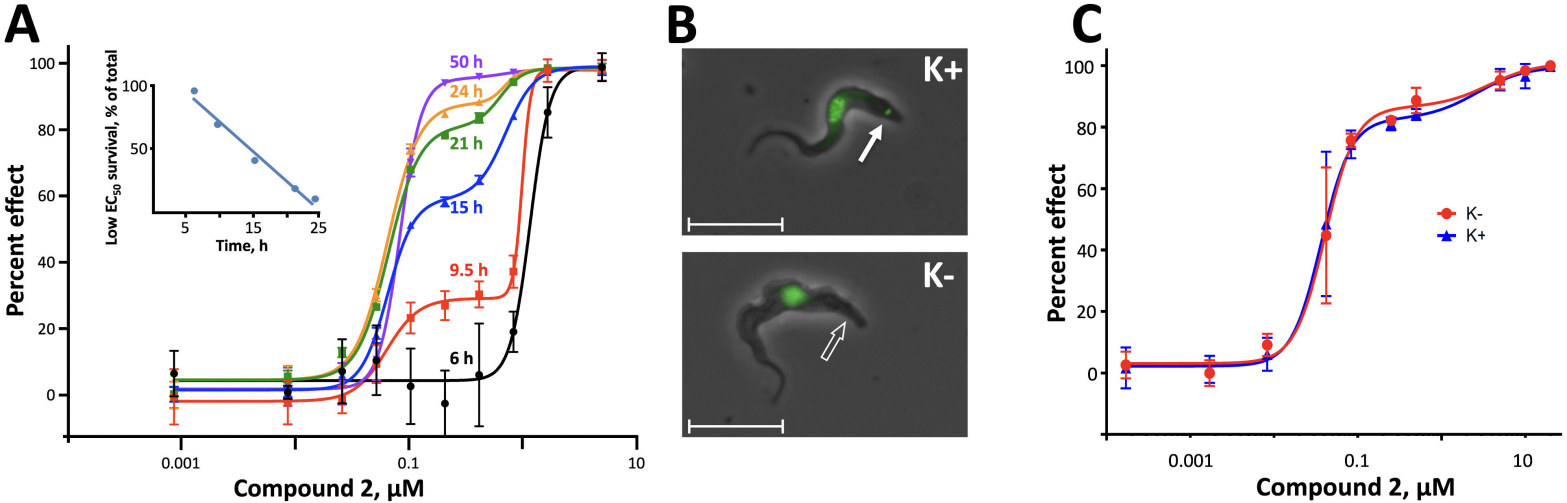
Time dependence of Compound **2** against *T. b. brucei* MiTat 1.2 and activity against akinetoplastic *T. b. brucei*. (**A**) Dose-response as a function of time. Low and high EC_50_ values were unchanged over time: 0.069 ± 0.008 and 0.83 ± 0.22, respectively. Depicted are M ± SD; some error bars are within the plotted symbols; all *R*^2^ values ≥0.995. (Inset) The proportion of low EC_50_ survivors falls over time; *y* = −4.6*x* + 120; *R*^2^ = 0.978. (**B**) Phase contrast image of *T. b. brucei* strain K164 (K*+*, upper panel) and its akinetoplastic mutant derivative, AK164 (K*−*, lower panel), overlaid with fluorescence images of SYBR Gold-stained cells. K+ parasites evidence both a nucleus and kinetoplast (solid arrow), whereas K− cells have only a nucleus. Hybridization studies of DNA isolated from akinetoplastic cells confirm the absence of kDNA (28). Bars, 10 µm (faint original size bars were replaced with thicker lines). (**C**) The activity of Compound **2** is comparable against parental kinetoplastic (blue) or akinetoplastic mutant (red) trypanosomes, in terms of low and high EC_50_ values (0.041 vs 0.037 µM and 3.7 vs 2.7 µM, respectively) and contributions of low EC_50_ (85% vs 82%). M ± SD; some error bars are within the plotted symbols; *R*^2^ values ≥0.966.

We explored several possible explanations for the observed biphasic dose-response curves against *T. b. brucei*. Intriguingly, the contributions of low and high EC_50_ fractions to overall efficacy evolved over time of drug exposure (Fig. 2A). Toxicity from a short exposure (6 h) to Compound **2** was attributable almost entirely to the high EC_50_ phase, but by 9.5 h, contribution of the low EC_50_ activity became evident and grew to 97% by 50 h. These results suggest that the more potent low EC_50_ component requires time to generate detectable cytotoxicity, whereas the less potent high EC_50_ component acts quickly. Of note, however, although their contributions to efficacy changed over time, the potency of each component remained constant.

#### Activity in akinetoplastic trypanosomes

Like nearly all eukaryotes, *T. b. brucei* have mitochondria and a mitochondrial genome, although most mitochondrial proteins are encoded in the nucleus. Mutants with no mtDNA (e.g., yeast petites and akinetoplastic trypanosomes) survive by a compensatory mutation in ATP synthase that maintains the essential transmitochondrial membrane potential (29). To test the possible contribution of mitochondrial RNA Pol 1 activity to the toxicity of Compound **2** against trypanosomes, we evaluated its effect against a parent kinetoplastic strain K164 (K+) and its akinetoplastic mutant AK164 (K−) (Fig. 2B) (28). Both strains evidenced a biphasic dose-response with indistinguishable EC_50_s and target ratios, effectively ruling out a contribution by impaired mitochondrial RNA Pol 1 activity (Fig. 2C).

#### Assay for DNA topoisomerase covalent adducts in *T. b. brucei*

Several antitrypanosomal agents bind avidly to DNA, either by intercalation (e.g., ethidium) or by minor groove binding (e.g., pentamidine), and poison the mitochondrial type II topoisomerase (30). To determine whether DNA damage mediated by intercalation and topoisomerase poisoning may contribute to the biphasic dose-response curves, we assayed for protein-DNA adduct formation. The class of topoisomerase inhibitors termed “poisons” acts by preventing the religation of DNA breaks created by the enzyme to allow strand passage (31). Such inhibitors are identified when transient catalytic intermediates are rapidly disrupted (e.g., by SDS denaturation) to forge covalent topoisomerase-DNA linkages that selectively precipitate with protein upon addition of KCl. Covalent linkage is confirmed by failure to precipitate after protease treatment. As expected for *T. b. brucei* (32), positive control topoisomerase poison etoposide doubled the metabolically labeled DNA in KSDS precipitates, and 80% of the precipitable label was protease sensitive (Table S2). However, after incubation with Compound **2**, KSDS-precipitable DNA was only marginally increased over no-drug controls and was not protease sensitive, indicating topoisomerase poisoning does not contribute meaningfully to Compound **2**’s dose-response in trypanosomes.

### *In vitro* combinations with Compound **2** against *T. b. brucei*

Previous reports indicated that combination of RNA Pol 1 inhibitors with other agents may be synergistic against tumor cells (33). We tested a number of drugs in combination with Compound **2** vs *T. b. brucei*, including topoisomerase inhibitors (camptothecin and etoposide), clinically used antitrypanosomals (eflornithine, melarsoprol, suramin, and pentamidine), and compounds reportedly synergistic with Compound **2** against cancer cells (17-AAG and temozolomide). All pairs were additive or antagonistic by isobologram analysis or combination index (CI) calculation (Table S3) (34).

### *In vitro* hollow fiber model pharmacokinetic-pharmacodynamic studies

**Fig 3:**
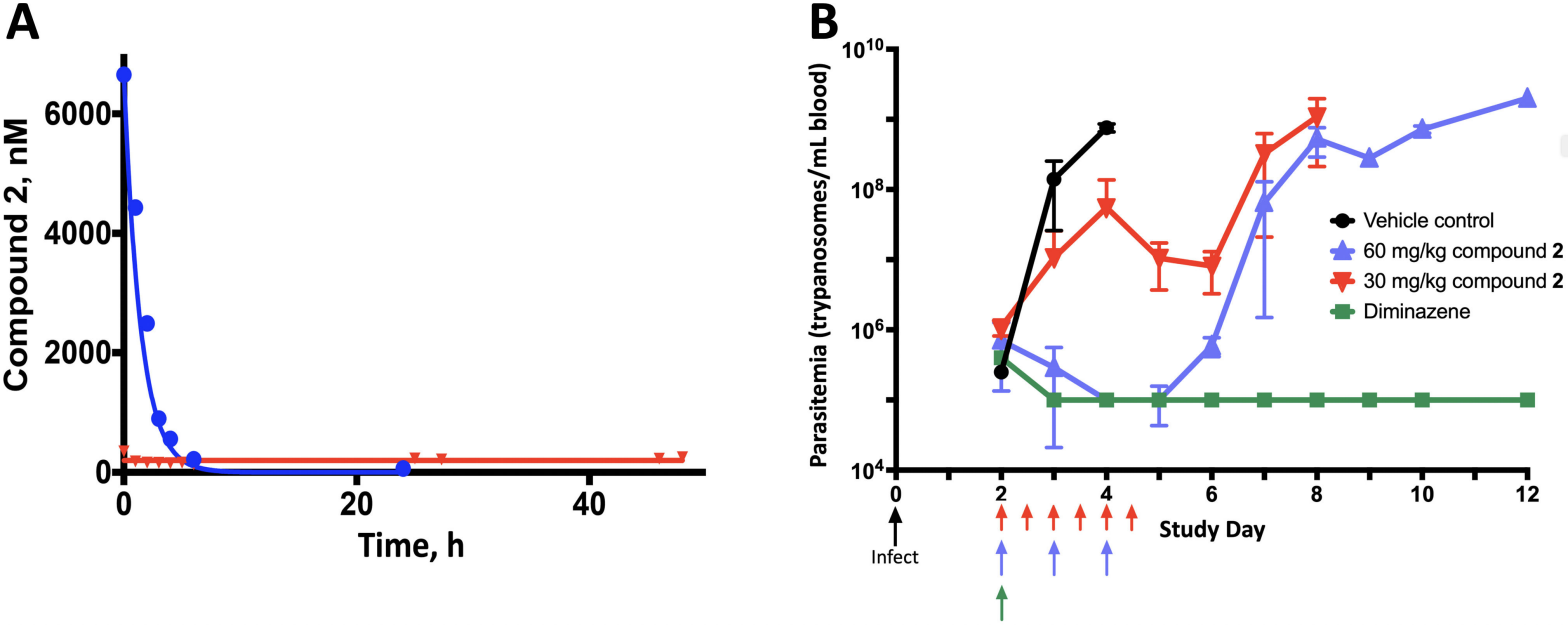
Compound **2** in vitro and in mice. (**A**) PK of Compound **2** in a hollow fiber cartridge experiment. The system was programmed to deliver AUC 9,600 nM•h over 48 h in widely different patterns: in one cartridge as a 200 nM constant concentration (red) and in a second cartridge as a bolus with peak 6,700 nM and *t*_1/2_ 1 h (blue). PK was obtained in timed samples of cartridge medium, via [^3^H]-labeled Compound **2** tracer. (Solid lines) Programmed kinetics; (symbols) observed kinetics. (**B**) Activity of Compound **2** against acute *T. b. brucei* MiTat 1.2 infection in mice. Animals were infected on study Day 0, then divided and treated in four cohorts as indicated, and monitored daily for up to 12 days. Parasitemia was obtained by light microscopy of tail snip blood samples (limit of detection, 10^5^ parasites/mL blood). Data are pooled results from two independent experiments, a total of eight or nine mice per dosing cohort. Parasitemia in vehicle controls (black) increased rapidly until the mice died or were sacrificed. By 2 days after starting diminazene aceturate (green, either 3.5 mg/kg daily for 3 days [two mice] or 10 mg/kg once [six mice]), parasitemia cleared and remained negative. As indicated, a total dose of 180 mg/kg Compound **2** over 3 days was given as 60 mg/kg once daily (blue) or 30 mg/kg every 12 h (red). Depicted counts are M ± SD.

Compound **2** was evaluated against *T. b. brucei* in pharmacokinetic-pharmacodynamic (PK-PD) cartridge experiments to determine the kinetic parameter (concentration or time) most responsible for antitrypanosomal activity (35, 36). The hollow fiber cartridge system deploys dynamic drug concentrations in a continuous flow of medium that more closely mimics *in vivo* conditions. Three cartridges were used for each experiment: one was a no-drug control, the other two provided a given dose and area under the concentration-time curve (AUC) as either a constant concentration (“infusion”) or as a short-lived high peak (“bolus”). The programmed kinetics were validated by radiolabeled tracer (Fig. 3A). Cells were harvested at the end of the AUC period and counted.

In all tested conditions, cytotoxicity was clearly superior when a given AUC of Compound **2** was deployed as a high peak with 1 h *t*_1/2_, rather than as a lower constant concentration (Table 2). Remarkably, this was true even in 48-h experiments in which drug concentrations in the bolus regimen fell below the EC_50_ by 8 h, and circulating medium was essentially drug-free for the final 40 h before harvest. Notably, the cytotoxicity of a given constant concentration of Compound **2** was systematically greater in a microtiter plate than in a cartridge, an effect seen previously and attributable to the beneficial effect of a constant flow of fresh medium (36).

**TABLE 2:** Kinetic driver of Compound **2** against *T. b. brucei* MiTat 1.2 in the hollow fiber cartridge system

Kinetics				Efficacy (percent effect vs untreated)			
AUC^*a*^ (nM•h)	Run time (h)	Bolus *C*_max_ (nM)	Infusion (nM)	Cartridge			Microtiter plate^*c*^
				Bolus	Infusion	B:I ratio^*b*^	
6,100	24	4,200	250	43	32	1.3	89
9,600	48	6,700	200	30	17	1.8	96
9,600	48	6,700	200	80	42	1.9	96

^*a*^ Selected AUCs were deployed against parasites as either a bolus with indicated *C*_max_ and *t*_1/2_ of 1 h or as a constant concentration over the entire run time (Fig. 3A). Higher AUC was designed to keep infusion concentration similar to that of the low AUC but with longer exposure time. AUCs of 9,600 nM•h are biological replicates.

^*b*^ Ratio of bolus:infusion (B:I) efficacies.

^*c*^ Efficacy at relevant infusion concentration, from dose-response curves after either 24- or 50-h drug exposure (Fig. 2A).

### Activity of compound **2** against *T. b. brucei* in mice

To assess antitrypanosomal activity *in vivo* and to determine whether it is concentration or time driven, we tested Compound **2** in a mouse model of acute peripheral infection with *T. b. brucei* MiTat 1.2 (Fig. 3B). An identical 3-day total dose of 180 mg/kg either was given as a single injection of 60 mg/kg/day or was dose fractionated as 30 mg/kg every 12 h. Both regimens evidenced antiparasitic activity relative to no-drug controls, but unlike positive control diminazene aceturate, neither regimen was curative (Fig. 3B). Nevertheless, the superior activity of single high daily doses of Compound **2**, compared to more frequent lower doses, was clearly evident. This finding, especially in the face of Compound **2**’s short plasma *t*_1/2_ of 2 h in mice (22), confirms the cartridge prediction of concentration-driven antiparasitic activity.

In an effort to obtain a cure for acute peripheral infections, we did a preliminary evaluation of escalating single daily doses of 75–600 mg/kg/day for multiple days (Fig. S2) but found that the selective toxicity of Compound **2** was insufficient to eradicate parasites without incurring significant morbidity and mortality. However, given the nearly 20-fold greater concentrations of Compound **2** in brain vs plasma (Fig. S1) and the need for new agents against CNS trypanosomiasis, we further piloted Compound **2** in a mouse model of late-stage infection with *T. b. brucei* TREU 667 (37). As in the acute infection model, Compound **2** alone (at 50 mg/kg/day for 6 days) had negligible activity against circulating parasites (Fig. S3, cohort D) and furthermore did not preclude invasion of the CNS that occurred before Day 11 (Fig. S3, cohort E). However, when given in conjunction with diminazene starting on Day 21, relapse from CNS infection was delayed (Fig. S3, cohort B vs A), suggesting there may be some (albeit insufficient) activity against established CNS infection.

## DISCUSSION

Constrained resources for the discovery and development of new drugs for parasitic infections have led to the notion of repurposing already-approved drugs for use in these diseases (38, 39). Successes include FDA-approved new indications for antifungal amphotericin B in visceral leishmaniasis or antibacterial doxycycline for malaria (40, 41). The dramatic efficacy of repurposed anticancer eflornithine in patients with African trypanosomiasis led to its characterization as a “resurrection drug” (42), and eflornithine became a drug of choice in trypanosome therapy. Based on the previously reported activity of RNA Polymerase 1 inhibitors against *T. b. brucei in vitro* (27), the unique requirement in trypanosomes of RNA Pol 1 for synthesis of VSG (13), the 20-fold CNS accumulation of RNA Pol 1 inhibitor leads in mice (Fig. S1), and the availability of a library of BMH-21 inhibitor analogs, we explored the possibility of repurposing this anticancer therapeutic strategy for use against African trypanosomes.

Initial *in vitro* cytotoxicity studies indicated that in *T. b. brucei*, as in mammalian cells, the structural allowances for BMH-21 analogs are quite limited. The four-ring benzopyridoquinazoline system (that mediates intercalation) is required, and alterations in the size or charge of the aliphatic side chain are restricted and sensitive even to relatively subtle steric differences. Given its potency against trypanosomes and the substantial body of information as an anticancer lead, our subsequent studies focused on stereoisomer LI-623 (Compound **2**). Of considerable interest was the finding that activity against *T. b. brucei* and tumor cells is biphasic (Fig. 1), which suggests the contribution of two possible targets. Precedent studies in mammalian cells and trypanosomes indicate that nuclear RNA Pol 1 is almost certainly a target for the low EC_50_ activity. We ruled out two possible candidates for high EC_50_ activity: mitochondrial RNA Pol 1 activity (Fig. 2C) or intercalation-sensitive DNA topoisomerases (Table S2).

Clues to the possible second target were obtained from the structure-activity series. Across several dozen analogs, there was a remarkable conservation in both the ratio of low-to-high EC_50_ fractions and in the ratio of EC_50_ values themselves: we could not dissect the targets apart on the basis of their structural requirements (which were similarly narrow) or their relative contributions to overall efficacy. Another insight into targets was obtained from the time course of dose-responses. The low EC_50_ component required time to have a detectable impact on trypanosomes, and once evident, the proportion of surviving cells fell at the fixed linear rate of 50% every 4.6 h (Fig. 2A, inset). These kinetics may reflect a combination of the 5.6-h median half-life of the proteome in bloodstream-form trypanosomes (43) in conjunction with the ~6-h doubling time of these cells that results in the dilution of essential components into daughter cells. Although a halt in RNA synthesis may be the most proximal target for Compound **2**, it is likely that the subsequent loss over time of rRNA and proteins is cytotoxic. In keeping with this is the previously reported reversibility of BMH-21 toxicity at 24 h of exposure, when downstream losses can still be tolerated (27).

Collectively, the available evidence suggests that DNA intercalation underpins both the low and high EC_50_ toxicities. One toxicity, sensitive but slow to become apparent, is the cumulative loss of rRNA and proteins; for at least 24 h, this is “static” (27). The second toxicity, which we could not identify, is 10-fold less sensitive but rapidly evident, and at high concentrations of BMH-21 is “cidal” (27). An intriguing possible explanation for the biphasic activity of BMH-21 analogs, which we did not explore, may stem from their recognized preferential (and dynamic) binding to non-canonical G-quadruplex nucleic acid secondary structures (44). Perhaps the biphasic dose-response is attributable to two possible modes of DNA binding (classical intercalation into a double helix plus non-canonical binding to G-quadruplex structures) that result in different downstream consequences for the cell.

Notably different for Compound **2** is the monophasic response of malaria parasites (Fig. 1C). Several factors could contribute to this. Although the extent of intercalation into DNA is generally independent of base composition, G-C specificity has been reported (45), and the G-C content in *P. falciparum* (<20%) is substantially lower than that in *T. b. brucei* (46.5%) or mice (42%) (46). Studies with BMH-21 against *P. falciparum* have revealed a highly variable effect on the abundance of individual RNA Pol 1-synthesized transcripts, which may also contribute to the contrasting shape of dose-response (47). Also dissimilar in these dose-response experiments is the doubling time of cells vs duration of exposure to test compounds. Trypanosomes and mammalian cells were exposed for more than four generations, which allows the low EC_50_ component to become apparent (Fig. 2A). *P. falciparum* was treated for 1.5 generations, perhaps not long enough (even in an asynchronous population) to allow the downstream cumulative loss of RNA and proteins to become evident.

We conducted several evaluations to assess further the therapeutic potential of Compound **2** in African trypanosomiasis. First was an *in vitro* analysis of PK-PD obtained with a hollow fiber model system we developed, as a means to improve the dosing of existing drugs and to inform the development of experimental agents. Key in this process is defining the component (concentration or time) of the dose-response AUC that drives antiparasitic efficacy. For over a dozen antitrypanosomals, we have used this *in vitro* system to define, unambiguously, the kinetic driver of efficacy (35, 36). The activity of Compound **2** against African trypanosomes *in vitro* is consistently concentration driven, independent of AUC or whether cells were treated for 24 or 48 h (Table 2). This finding was confirmed in a 3-day study of mice infected with *T. b. brucei*, in which the same total dose was more efficacious when given as a large daily injection rather than smaller ones every 12 h (Fig. 3B). Somewhat counterintuitively, in this experiment, the high concentration AUC is superior despite the 2-h plasma *t*_1/2_ of Compound **2** in mice (22). These findings indicate that structural modifications to prolong plasma half-life would not meaningfully improve activity. To exploit the concentration-driven activity in mice, we evaluated escalating doses of single daily injections but found that cure was accompanied by significant morbidity and mortality (Fig. S2). Finally, given the 20-fold accumulation of Compound **2** in mouse brain tissue (Fig. S1), we conducted a pilot study to evaluate its activity in a mouse model of CNS infection. Results suggested that CNS parasites were affected but not eradicated (Fig. S3).

Although inhibition of RNA Pol 1 is a novel and effective target in African trypanosomes (perhaps including *T. congolense* or *T. vivax*, in addition to the *T. bruce*i subspecies reported here), these studies indicate that the benzopyridoquinazoline-based inhibitors have a narrow therapeutic index and that a different and more selective pharmacophore would be required for further development toward clinical applications. Possible candidates include G-quadruplex-binding small molecule scaffolds, several of which have selective antitrypanosomal activity (reviewed in reference 48). This work also highlights the predictive value of an *in vitro* hollow fiber model system to characterize experimental antitrypanosomal PK-PD.

## MATERIALS AND METHODS

### Cell cultivation

In this report, all studies with trypanosomes were conducted with bloodstream-form *T. brucei subspecies*, including (i) *T. b. brucei* MiTat 1.2 strain 427 (ATCC, used in most studies); (ii) *T. b. gambiense* from Dennis Grab (Uniformed Services University; see reference 49; Fig. 1B only); or (iii) *T. b. brucei* isogenic paired strains K164 (wild-type parent) and AK164 (its akinetoplastic mutant) from Kenneth Stuart (Seattle Children’s Research Institute; see reference 28; Fig. 2B and C only). *T. b. brucei* MiTat 1.2 and *T. b. gambiense* were cultured in phenol red-free HMI-9, 10% fetal bovine serum (Sigma-Aldrich) and 10% Serum Plus (Sigma-Aldrich) (35, 36); K614 and AK614 were maintained in conditioned medium. Asynchronous *P. falciparum* NF54 HOX (ATCC) was grown in phenol red-free RPMI with 4.4 µM hypoxanthine, 50 mM HEPES, and 27 mM NaHCO_3_, in O^+^ erythrocytes and 0.5% AlbuMAX II (Life Technologies), at 1.2% or 2.4% hematocrit and 0.25%–6% parasitemia (50, 51). Erythrocytes were obtained weekly from healthy donors under an Institutional Review Board-approved protocol. L1210 murine lymphoblastic leukemia cells (ATCC) were cultured in phenol red-free RPMI-1640 supplemented with 15% fetal bovine serum (vol/vol). All cell lines were maintained in continuous exponential growth at 37°C in a humidified incubator under 5% CO_2_ in air. L1210 cells and motile trypanosomes were counted by light microscopy with hemocytometer; malaria parasites were counted in SYBR Gold-stained thin smear by fluorescence microscopy.

### Test compounds

LI-623 (Compound **2**) was obtained from Wuxi Apptec Co. BMH-21 (**1**), and its analogs were synthesized as described previously (24, 26); their purity was evaluated by liquid chromatography/mass spectrometry and ^1^H nuclear magnetic resonance spectroscopy. BMH-21 (CID: 3508054) and LI-623 (BOB-42, Compound **2**) (CID: 90644360) are deposited in PubChem.

Indicated are sources for diminazene aceturate, etoposide and camptothecin (Sigma-Aldrich), suramin (Cayman Chemicals), pentamidine (Fresenius Kabi), temozolomide (Tokyo Chemical Industry), melarsoprol (US CDC), and eflornithine and 17-AAG (NCI DTP). Compounds were dissolved in DMSO (Sigma-Aldrich) or a solution of 0.1 M potassium phosphate; unless indicated otherwise, the final concentration of DMSO in cell cultures did not exceed 0.2%. Drugs for animal testing were dissolved in sterile phosphate-buffered saline (PBS) immediately prior to dosing.

### Cytotoxicity assays

Activity against *T. b. brucei* MiTat 1.2 or L1210 cells was evaluated by the acid phosphatase method (52). Briefly, 10^5^/mL trypanosomes or 7 × 10^4^/mL L1210 cells were incubated 24 or 48 h, respectively, with test compound in quadruplicate in flat-bottom 96-well plates (Falcon, Denville) before lysis with 0.1 vol 20 mg/mL *p*-nitrophenylphosphate, 1% vol/vol Triton X-100, 1 M acetic acid, pH 5.5. Lysates were incubated (37°C, 3–6 h) prior to reading (*λ* = 405 nm, SpectraMax Plus 384). Percent effect was calculated based on the absorbance of compound-free and 100% effect controls. Paired inhibitors were evaluated by checkerboard assays entailing 8 × 8 combinations, evaluated by isobologram and CI methods (34).

Antimalarial activity was based on metabolic incorporation of radiolabeled hypoxanthine (50, 53). Parasites (0.25% parasitemia and 1.2% hematocrit) in 96-well plates (Costar) were incubated with varying concentrations of compound, each in quadruplicate. At 48 h, [^3^H]hypoxanthine (PerkinElmer NET0177, 14 Ci/mmol) was added to each well (final concentration 0.25 µCi/mL), and at 72 h, cells were harvested onto GF/C glass fiber filters (Brandel), lysed, and washed, and incorporated radiolabel was measured (Beckman Coulter LS6500). EC_50_s were obtained with GraphPad Prism (see “Statistics and graphs,” below).

### Photomicrographs

K614 or AK614 *T. b. brucei* cells in culture were pelleted (400 × *g*, 10 min, room temperature), resuspended in an equal volume of medium, spotted on glass slides, smeared, air dried, and fixed in 100% MeOH. Preparations were stained with SYBR Gold and visualized (Zeiss AxioImager M2 fluorescence microscope equipped with oil-immersion Zeiss Plan Apo63x/NA 1.4 objective and Hamamatsu ORCA-R2 camera), and images were acquired using Volocity 6.3.1 software (Quorum Technologies Inc, Puslinch, ON, Canada).

### *In vitro* PK-PD of compound **2** against trypanosomes

Activity against *T. b. brucei* MiTat 1.2 in a hollow fiber model system was assessed as described previously (35, 36). Autoclave-sterilized apparatus, including C2025 cartridges (FiberCell Systems), reservoirs, platinum-cured silicone tubing (Masterflex, Cole-Parmer), and 2-Stop 1.52 mm ID Pump Tubing (IDEX Health & Science), were assembled with peristaltic pump (IPC Pump Ismatech) in a biosafety cabinet and maintained in a 37°C, 5% CO_2_ incubator. Medium was pumped continuously through the system at 14 mL/h. Drug concentrations were determined by tracer amounts of [^3^H]LI-623 (2.6 Ci/mmol, Moravek Inc). PK was first established in the absence of parasites by iterative adjustment of programmed kinetics to compensate for the observed 96% non-specific binding, then final kinetics were validated. For PK-PD, cells were introduced at 10^5^/mL; inoculum culture was maintained in parallel flasks. Cells were harvested at 24 or 48 h, and activity was determined by comparing live parasite counts in drug-treated vs untreated controls.

### Compound **2** against trypanosomes in mice

Six-week-old male or female CD1 mice (Charles River Laboratories) were inoculated intraperitoneally with 10^5^ *T. b. brucei* MiTat 1.2 strain 427 (ATCC) on Day 0, and parasitemia was monitored by microscopic exam of tail snip blood on duplicate counts of replicate samples. At a parasitemia of ~10^6^/mL, animals were divided into cohorts and dosed intraperitoneally with 100 µL PBS or compound solution prepared immediately prior to use. Parasitemia was monitored daily, and mice were euthanized for evident distress or for counts ≥5 × 10^8^/mL. Parasites were quantified via hemocytometer or by counts in 20 fields at 400× magnification. From standard curves, trypanosomes/mL = (1.9 × 10^4^) × count in 20 fields.

### Statistics and graphs

Cytotoxicity data were analyzed in GraphPad Prism (6.0 or 10.2.0) by non-linear fit with (biphasic + True EC_50_) parameters to provide an EC_50_ and fractional contribution for each phase of the curve, as well as overall EC_50_ and *R*^2^ values. Structural analogs were compared by unpaired, two-tailed *t*-test of their overall EC_50_s. All data are expressed as mean (M) ± SD.

## Data Availability

No nucleotide or amino acid sequences, microarray data, protein structures, gene expression data, or Mycobank data were generated in this project.

## SUPPLEMENTAL MATERIAL

Supplemental material aac.01492-25-s0001.pdf Tables S1 to S3; Fig. S1 to S3.

ASM does not own the copyrights to Supplemental Material that may be linked to, or accessed through, an article. The authors have granted ASM a non-exclusive, world-wide license to publish the Supplemental Material files. Please contact the corresponding author directly for reuse.
