# Supplementary material for: RNA Polymerase 1 inhibitors against African trypanosomes in vitro and in mice
Source: Antimicrob Agents Chemother. 2026 Mar 27;70(5):e01492-25. doi: 10.1128/aac.01492-25 (PMC13140157; doi:10.1128/aac.01492-25)
Supplement: Supplemental material — Tables S1 to S3; Fig. S1 to S3. [file aac.01492-25-s0001.pdf]

TABLE S1 Cytotoxicity of additional RNA Polymerase I inhibitors against *T. brucei* MiTat 1.2 *in vitro*

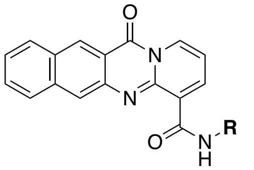

Scaffold A

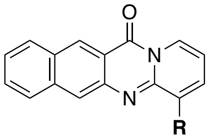

Scaffold C

Compound	Scaffold	R	EC_{50} (µM)[a]
**1** (BMH-21)	A	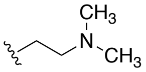	0.11
**13**	A	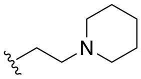	0.37[b]
**14**	A	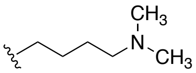	0.42
**15**	A	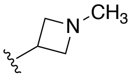	0.52 [b]
**16**	A	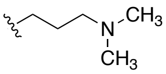	0.78 [b]
**17**	A	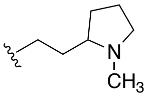	2.2
**18**	A	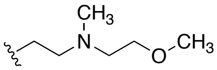	3.9
**19**	A	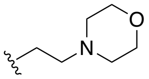	4.4
**20**	A	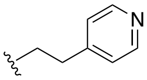	58% at 0.5
**21**	A	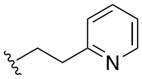	57% at 0.5
**22**	A	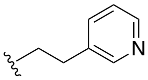	41% at 0.5
**23**	A	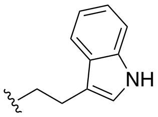	>5
**24**	A	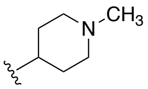	65% at 0.5

**25**	A	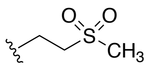	>0.5
**26**	A	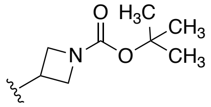	>5
**27**	C	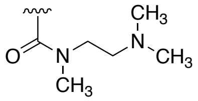	>5
**28**	C	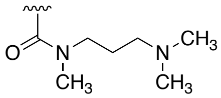	>5
**29**	C	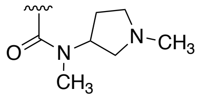	>5
**30**	C	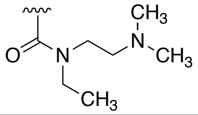	>5
**31**	C	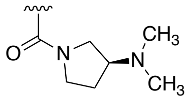	>5
**32**	C	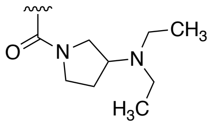	>5
**33**	C	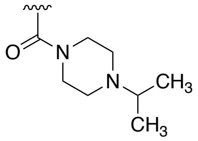	>5
**34**	C	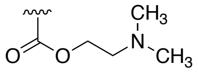	>5

[a]Compounds were tested to limit of solubility. Overall EC_{50}s were obtained from biphasic curve fit. Unless indicated otherwise, data from one experiment in quadruplicate. Excluding Compound **1** (whose data are included in Table 1), for all dose-responses, R^2 ≥0.994; CV for all data points ≤9.2% and M ± SD for all data point CVs was 1.6% ± 0.7% (n = 121).

[b]Data from two or more biological replicates in quadruplicate

TABLE S2 Protein-DNA covalent adduct formation for *T. b. brucei* MiTat 1.2

**Addition**	**KSDS-precipitable Counts** (percent of total incorporation[a])		
	**Baseline**	**After protease digestion**[b]	**Protease sensitivity**
None	30	ND	–
DMSO, 1%	30	ND	–
Etoposide, 100 μM	62	12	81%
Compound **2**, 100 μM	35	37	none

[a]Total incorporation = 181,600 dpm precipitated by TCA from 10^6 cells after 1 hr labeling

[b]ND, not determined

Covalent protein-DNA adduct formation in trypanosomes was assessed as described previously (1,2). *T. b. brucei* MiTat 1.2 (2 x 10^6/mL) was metabolically labeled for 1 h (37 ºC, 3 h) with [methyl-^{3}H]thymidine (PerkinElmer, 100 μCi/mL; 20 Ci/mmol), washed and resuspended in IMDM at 2 x 10^6/mL. To establish total incorporation of label, quadruplicate samples of radiolabeled cells were applied to Whatman paper, washed with cold 5% trichloroacetic acid, then 95% ethanol, and counted (CytoScint fluid, MP Biomedicals; LS6500 counter, Beckman Coulter). Remaining radiolabeled cells were aliquoted and treated (37 ºC, 15 min) in quadruplicate with medium, 1% DMSO, 100 μM etoposide, or 100 μM Compound **2,** and lysed with 2.5% SDS, 0.2 mg/mL sheared calf thymus DNA, 10 mM EDTA. Half the samples were then digested (50 ºC, 1h) with 3.4 mg/mL Proteinase K. Protein and covalent radiolabeled DNA-protein complexes were precipitated (on ice, 1 h) by addition of 70 μL 1M KCl and captured by filtration through Whatman filter paper prewetted with wash buffer (10 mM Tris-HCl pH 8, 1 mM EDTA, 100 mM KCl). Precipitates were washed thrice with wash buffer and counted.

TABLE S3 *In vitro* interaction of Compound **2** paired with other agents against *T. b. brucei* MiTat 1.2

**Partner**	**Combination Index at EC_{50}[a] (M ± SD)**	**Number of evaluable pairs**
Camptothecin	1.31 ± 0.19	6
Etoposide	1.17 ± 0.17	4
Eflornithine	1.12 ± 0.10	5
Melarsoprol	1.18 ± 0.13	4
Suramin	1.33 ± 0.37	6
Pentamidine	1.49 ± 0.60	6
17-AAG	1.06 ± 0.13	4
Temozolomide	1.21 ± 0.20	7

[a]Data from 8 x 8 checkerboard assays were plotted to obtain families of dose-response curves and their resulting EC_{50} isobolograms. CI was calculated for each evaluable EC_{50} pair. CI of 1 is additive; CI < 1 is synergistic; CI >1 is antagonistic.

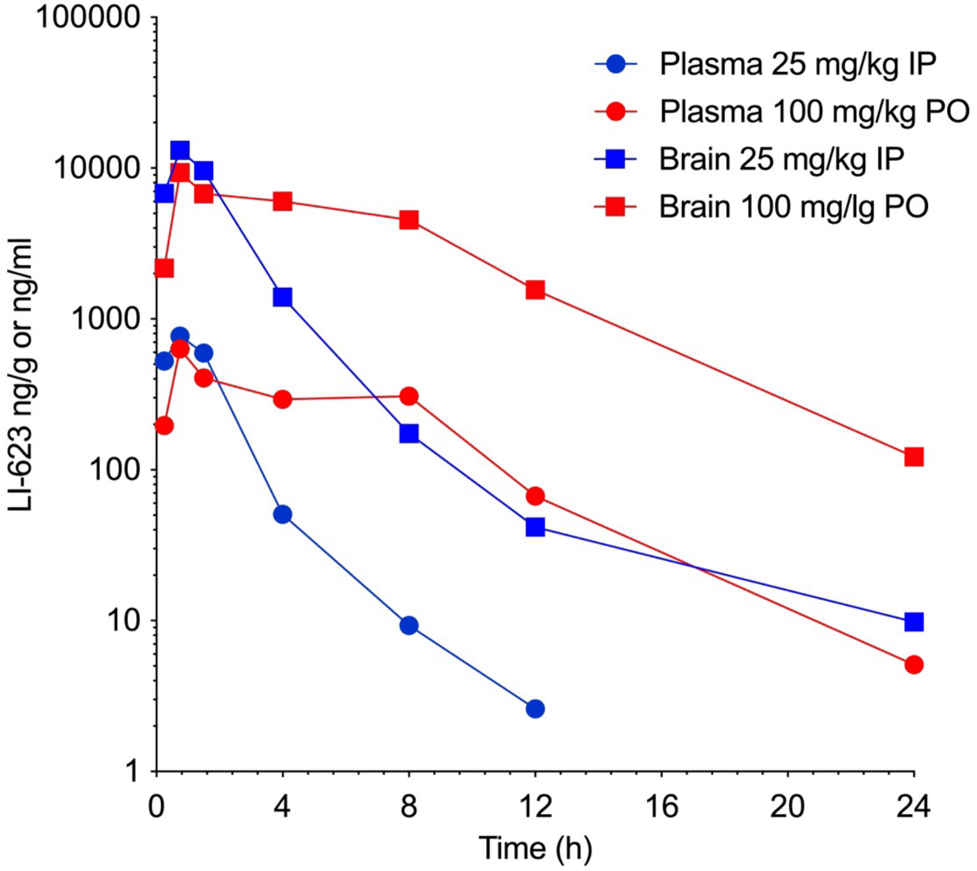

Fig S1 Tissue distribution of Compound **2** in mice. A biodistribution study was conducted using a single 25 mg/kg intraperitoneal or 100 mg/kg oral dose of **2** dissolved in 0.2 M phosphate buffer. Male FVB mice (Charles River) (n = 3 for each timepoint) were euthanized at 15 min, 45 min, 1.5 h, 4 h, 8 h, 12 h, and 24 h, blood was collected using cardiopuncture and brains were dissected, washed in cold PBS and snap frozen. The samples were analyzed by LC-MS/MS (Shimadzu Nexera X2 UHPLC with Sciex 4500 Triple Quad Mass Spec) at the Analytical Pharmacology Facility at Sidney Kimmel Comprehensive Cancer Center. The study was conducted according to the animal experimentation permit of the Animal Care and Use Committee at the Johns Hopkins University.

A

Expt number	Mice	Treatment			
		Drug	Dose (mg/kg/d)	Days	Total (mg/kg)
1 & 2	4+3	Vehicle	–	1	–
1	2	Diminazene	10	1	10
1	3	Compound **2**	100	1-5	500
1	3	Compound **2**	100	1-4	400
2	1	Compound **2**	100	1-3	300
2	2	Compound **2**	75	1-4	300
2	2	Compound **2**	60	1-5	300
2	1	Compound **2**	60	1-4	240

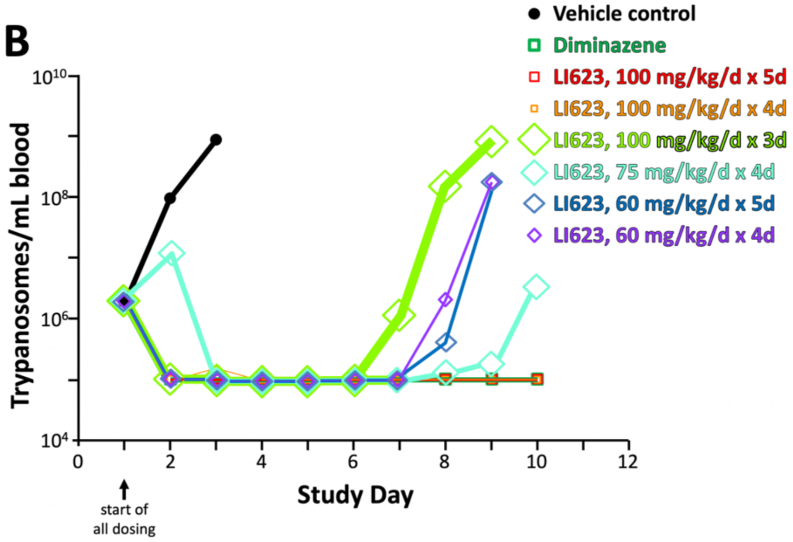

FIG S2 Pilot studies of single daily doses of Compound **2** against acute *T. b. brucei* MiTat 1.2 strain 427 infection in mice, as described in Methods. **(A)** Animals were infected with 10^5 trypanosomes, divided and treated in cohorts as indicated, and monitored daily for up to 10 days. **(B)** Parasitemia in negative control vehicle-treated mice increased rapidly until the animals died or were sacrificed (*black circle*). In positive controls, by one day after a single dose of 10 mg/kg diminazene aceturate, parasitemia cleared and remained negative (*dark green square*). Compound **2** at 100 mg/kg once daily for five or four days (500 or 400 mg/kg total dose; *red or orange squares*) was apparently curative, but the mice died or were sacrificed on days 6 - 10 because of drug toxicity. The remaining regimens, for a total dose of 300 mg/kg or less (*diamonds*), provided only transient clearance of parasitemia; deaths in these animals were attributable to parasitemia. Parasitemia was obtained by light microscopy of tail snip blood samples (limit of detection, 10^5 parasites/mL blood). *Symbols,* average of duplicate counts on duplicate blood samples from number of mice listed in Panel A. Data are from two independent experiments.

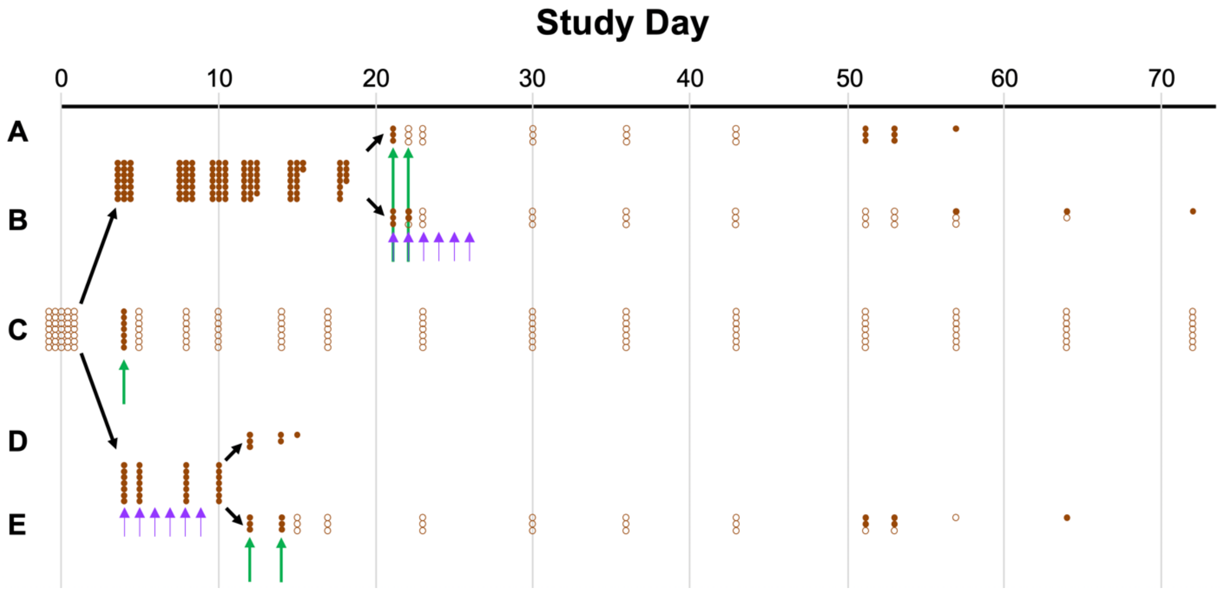

Fig S3 Pilot study of Compound **2** in the *T. b. brucei* TREU 667-mouse-diminazene model of late stage central nervous system (CNS) trypanosomiasis. In this model (3), circulating trypanosomes reach the CNS 7-21 d after start of infection. To create a CNS-only infection, on day 21 peripheral parasites are eliminated with diminazene aceturate (which does not penetrate into CNS) prior to start of experimental therapy. Failure against CNS parasites is evidenced by the re-appearance of trypanosomes in blood. This experiment was conducted in 6 week old female CD1 mice (Charles River). *T. b. brucei* TREU 667 (frozen stabilate, kindly provided in 1988 by F. W. Jennings at the University of Glasgow) was thawed and inoculated intraperitoneally into a single mouse. On study day zero blood was harvested from the anesthetized mouse by cardiac puncture, diluted to 10^5 trypanosomes/mL (sterile phosphate-buffered saline with 1% glucose) and aliquoted to infect thirty-five mice with 10^4 parasites/100 µL/mouse by ip injection (*open circles*). On day 4 each mouse was verified parasitemic (*filled circles*) at 10^6/mL by light microscopy of tail snip blood; animals were divided into three cohorts (AB, C, and DE) as indicated and treated with no drug (cohort AB), a single intraperitoneal dose of diminazene aceturate 40 mg/kg (*green arrow*; cohort C), or Compound **2** 50 mg/kg daily for six days (*purple arrows*; cohort DE). In Cohort C parasites were undetectable by day 15 (*open circles*) and remained so until mice were sacrificed at day 72, indicating diminazene eliminated peripheral parasites before CNS invasion occurred. Compound **2**-treated animals in cohort DE remained parasitemic, and on day 10 were divided into two groups. To assess activity of **2** against peripheral parasites, Cohort D (3 mice) had no further treatment and all mice died by day 15. Cohort E (3 mice) was further treated with diminazene and although peripheral parasitemia was cleared, by day 51 relapses appeared and all mice subsequently died. This indicates that early treatment with **2** does not prevent CNS invasion. Cohort AB was untreated for 21 days, by which time 70% of the mice had died. On day 21 the remaining six animals were divided into two groups. Cohort A was treated with diminazene alone and parasitemia cleared but all three mice relapsed, verifying that trypanosomes had invaded the CNS before day 21. Cohort B was additionally dosed with Compound **2** 50 mg/kg daily for six days. All three of these mice also relapsed, but the interval from treatment to failure was longer than that after diminazene alone in cohort A, which suggests **2** may have partial activity against CNS parasites.

REFERENCES

1. Shapiro TA, Englund PT. 1990. Selective cleavage of kinetoplast DNA minicircles promoted by antitrypanosomal drugs. Proc Natl Acad Sci U S A 87:950-4.

2. Bodley AL, Shapiro TA. 1995. Molecular and cytotoxic effects of camptothecin, a topoisomerase I inhibitor, on trypanosomes and *Leishmania*. Proc Natl Acad Sci U S A 92:3726-30.

3. Jennings FW, Whitelaw DD, Urquhart GM. 1977. The relationship between duration of infection with *Trypanosoma brucei* in mice and the efficacy of chemotherapy. Parasitology 75:143-53.
